# Being Tired or Having Much Left Undone: The Relationship Between Fatigue and Unfinished Tasks With Affective Rumination and Vitality in Beginning Teachers

**DOI:** 10.3389/fpsyg.2022.935775

**Published:** 2022-07-04

**Authors:** Gerald M. Weiher, Yasemin Z. Varol, Holger Horz

**Affiliations:** Department of Educational Psychology, Institute of Psychology, Goethe University Frankfurt, Frankfurt, Germany

**Keywords:** affective rumination, fatigue, unfinished tasks, vitality, cyclical processes

## Abstract

The present diary study was conducted for the purpose of bridging and integrating empirical research on the antecedents and consequences of work-related ruminative processes in the evening. Based on the control theory, unfinished tasks and fatigue in the afternoon were considered as antecedents of affective rumination, while vitality was investigated as the outcome observed in the next morning to test for cyclical processes. During a 5-day diary study (including 3 weekdays and the weekend), 74 beginning teachers completed three diary entries per day. A total of 795 diary entries were obtained. Using multilevel structural equation modeling, the study supported that both fatigue and unfinished tasks explained unique shares of variance of affective rumination in the evening at the between- and within-person levels. Furthermore, affective rumination mediated the relationship between unfinished tasks and vitality as well as fatigue and vitality. However, this only held true at the between- and not the within-person level, as neither affective rumination nor fatigue and unfinished tasks predicted the following morning’s vitality at this level. The results offer insights into the antecedents of affective rumination and add to extant research on the negative consequences of affective rumination considering vitality as an outcome.

## Introduction

Negative work-related thoughts during non-work time hinder recovery from work-related stress (Wendsche et al., 2021; Jimenez et al., 2022). Affective rumination, for example, has been linked to sleep impairments (Syrek et al., 2017), higher fatigue, and lower levels of vigor in the evening (Minnen et al., 2021) as well as health complaints and emotional exhaustion (Firoozabadi et al., 2018a) in cross-sectional, longitudinal, and diary studies. A recent meta-analysis further confirmed that affective rumination, which is categorized as “work-related thoughts and associated feelings,” is related to lower wellbeing and higher health complaints (Jimenez et al., 2022). Therefore, it is crucial to understand what leads to affective rumination to effectively prevent its occurrence.

Several models have tried to explain why affective rumination occurs (c.f. Wendsche et al., 2021); however, the exact mechanism remains unclear. The *stressor*-*detachment model* (Sonnentag and Fritz, 2015) offers one approach, which can be further connected (cf. Wendsche et al., 2021) to *the recovery paradox* phenomenon (Sonnentag, 2018). The original stressor-detachment model refers to psychological detachment, which refers to being mentally disengaged and distanced from work during non-work time (Sonnentag and Fritz, 2015). The model proposes that work-related stressors reduce psychological detachment during leisure time and increase the possibility of negative work-related thoughts, which subsequently increase strain reactions (Sonnentag and Fritz, 2015). The recovery paradox expands this assumption and contends that work-related stressors can cause higher negative activation, higher fatigue, and lower levels of energy resources. This would lead to negative work-related thoughts and difficulties in controlling them (Sonnentag, 2018). Recent longitudinal studies with two to three measurement points supported the notion that higher levels of fatigue or exhaustion might impair mental distancing from work (Sonnentag et al., 2014; Schulz et al., 2020).

Martin and Tesser (1996) developed a significant model, which assists in explaining why work-related thoughts occur during non-work time. It is connected to motivational or control theory approaches (cf. Watkins, 2008) and has been recently described as the *self-regulation model of ruminative thoughts* (Wendsche et al., 2021). According to this model, ruminative thoughts occur in the presence of lower than anticipated progress in attaining a goal. Thus, discrepancies between one’s present state and one’s goal lead to ruminative thoughts (Martin and Tesser, 1996). Studies building on this model focused on unfinished tasks as a proxy for discrepancies between an individual’s present state and goal attainment. Their findings showed that having unfinished tasks before the weekend was associated with higher affective rumination over the weekend (Syrek and Antoni, 2014; Syrek et al., 2017; Weigelt et al., 2018).

While previous studies have tested these models and provided important insights into work-related thoughts during non-work time, the recovery paradox phenomenon and the self-regulation model of ruminative thoughts are yet to be integrated and tested simultaneously. This is crucial because it remains unknown whether unfinished tasks, higher fatigue, or both trigger ruminative thoughts during non-work time. This makes it difficult to draw theoretical and practical conclusions regarding affective rumination during non-work time. Furthermore, the daily effects of unfinished tasks and fatigue remain unassessed. This is crucial because fatigue, unfinished tasks, and work-related thoughts may vary between days in accordance with work-related changes (Sonnentag and Fritz, 2015; Sonnentag et al., 2021; Wendsche et al., 2021). Therefore, day-level within-person fluctuations are crucial for testing the effects outlined in the above-mentioned theories.

To elucidate the differential role of unfinished tasks and fatigue in predicting affective rumination in the evening, we conducted a daily diary study. We argue that the aforementioned theoretical assumptions on what causes affective rumination can be integrated using motivational and control theory approaches (cf. Martin and Tesser, 1996; Watkins, 2008). Additionally, the study adds to the scant research (Firoozabadi et al., 2018b; Wach et al., 2020) focusing not only on the effect of affective rumination on fatigue in the evening and on sleep (e.g., Syrek et al., 2017; Minnen et al., 2021), but also on next-day functioning with respect to next-day vitality. Vitality refers to a “positive feeling of aliveness and energy” (Ryan and Frederick, 1997, p. 529) and having energetic resources as a function of one’s fatigue level (Ryan and Frederick, 1997; Fritz et al., 2011). This allows for testing cyclical processes (cf. Sonnentag et al., 2021), as fatigue in the afternoon can cause affective rumination, which may subsequently affect vitality the following morning.

Our study contributes to extant literature in several ways. First, the assumptions of the recovery paradox (Sonnentag, 2018) and the self-regulation theory of ruminative thoughts (Martin and Tesser, 1996) are considered simultaneously in testing unfinished tasks and fatigue as predictors of affective rumination. Therefore, the existing literature and theoretical assumptions regarding why work-related ruminative thoughts occur in the evenings are bridged and integrated. Second, testing the mediation effect of affective rumination in the relationship between fatigue and unfinished tasks, and vitality the next morning, allows for testing cyclical or reciprocal processes. This is because affective rumination may be affected by fatigue after work and affects vitality before work. To test this assumption, multiple measurement points and testing for within-person and between-person associations are required (cf. Sonnentag et al., 2021). This is due to the association of daily fatigue and daily affective rumination with vitality, which may differ when compared to the associations of general, overall fatigue and affective rumination. By using three measurement points per day, the present study allowed for testing the cyclical or reciprocal processes mentioned and evaluating whether states immediately after work are associated with the state immediately before work the next morning. Third, our study adds to the scant research (e.g., Kinnunen et al., 2019; Minnen et al., 2021) focusing on the effect of affective rumination on more positive connoted forms of outcome as indicators of good wellbeing (Minnen et al., 2021). Specifically, we considered vitality as the opposite and positive connoted equivalent of the state of feeling fatigued. Furthermore, until date, most studies have focused either on the effect of rumination on wellbeing or on the sleep quality of employees over longer time frames, such as after several months or a weekend (e.g., Syrek et al., 2017; Firoozabadi et al., 2018b; Weigelt et al., 2018; Kinnunen et al., 2019; Minnen et al., 2021). The present study contributes to the research by specifying the time frame of the association of affective rumination with wellbeing on the next day, which has rarely been explored (e.g., Firoozabadi et al., 2018a; Wach et al., 2020). Therefore, the existing literature was replicated and extended.

### Affective Rumination

Rumination was originally defined as “*a class of conscious thoughts that revolve around a common instrumental theme and that recur in the absence of immediate environmental demands requiring the thoughts*” (Martin and Tesser, 1996, p. 7). Although this definition used the term *thoughts*, the authors emphasized that not only verbal content, but also emotions and images, formed part of rumination (Martin and Tesser, 1996). Even though Martin and Tesser (1996) contended that rumination was not necessarily considered negative, it was often related to depression and negative outcomes (Nolen-Hoeksema et al., 2008; Watkins and Nolen-Hoeksema, 2014). Cropley and Zijlstra (2011) introduced the concept of work-related rumination (WRR), in which affective rumination is one of the facets of WRR. It is defined as “a cognitive state characterized by the appearance of intrusive, pervasive, recurrent thoughts, about work, which are negative in affective terms” (Cropley and Zijlstra, 2011, p. 10). It refers to negative emotional reactions to work-related thoughts (Weigelt et al., 2019). It is also associated with recovery experiences and outcomes above the positively valenced form of work-related rumination (Querstret and Cropley, 2012; Syrek et al., 2017; Wendsche et al., 2021; Jimenez et al., 2022). Weigelt et al. (2019) demonstrated that affective rumination is a unique concept associated with higher burnout and lower vitality. Affective rumination refers to a state of work-related thoughts during non-work time (Cropley and Zijlstra, 2011). However, it has been established as a state (within-person variance) as well as varying between individuals (between-person variance), as seen in cross-sectional and several diary studies (e.g., Cropley et al., 2012; Querstret and Cropley, 2012; Syrek et al., 2017; Firoozabadi et al., 2018a,b; Wach et al., 2020). As affective rumination can be conceptualized on both levels and is highly related to exhaustion levels (see below), we thus focused on affective rumination despite other work-related thought conceptualizations.

Research on work-related thoughts during non-work time often focuses on psychological detachment, which is defined as the absence of work-related thoughts during non-work time (Sonnentag and Fritz, 2015). Several meta-analyses provided evidence that lower levels of psychological detachment are related to lower levels of wellbeing indicators, such as life satisfaction or sleep as well as higher levels of fatigue (cf. Wendsche and Lohmann-Haislah, 2017; Bennett et al., 2018; Headrick et al., 2019; Steed et al., 2021). Recently, Jimenez et al. (2022) conducted a meta-analysis focusing on the content of work-related thoughts during non-work time. Affective rumination as defined above was included in the category of negative work-related thoughts and feelings (NWRTFs). Jimenez et al. (2022) showed that NWRTFs were associated with lower levels of task performance, job satisfaction, and work engagement, and higher levels of negative affectivity, burnout, and health complaints. Jimenez et al. (2022) concluded that NWRTFs had the strongest relationship with outcomes (e.g., burnout and health complaints) compared to other forms of work-related thoughts, which may be due to the affective strain experiences of work-related thoughts. Even though the meta-analysis did not differentiate between affective rumination and other conceptualizations of NWRTFs, the findings were consistent with the aforementioned studies. Therefore, it is feasible to suggest that affective rumination is related to employees’ negative affectivity, exhaustion, and health complaints.

### Prediction of Affective Rumination

There are several theoretical assumptions regarding why negative work-related thoughts occur during non-work time (Wendsche et al., 2021). As previously mentioned, Martin and Tesser (1996) contended that rumination is experienced in situations of unattained goals or unexpected (low) goal progress. Translated to work context, an employee would ruminate on the unattained tasks at hand (Martin and Tesser, 1996; Syrek et al., 2017; Wendsche et al., 2021). Rumination occurs until the discrepancy is overcome, or the person abandons the goal (Martin and Tesser, 1996; Wendsche et al., 2021). This model is often coupled with the “Zeigarnik effect” (Zeigarnik, 1938; Wendsche et al., 2021), specifically, that unfinished or interrupted tasks would lead to better retention of the unfinished tasks. For example, Syrek et al. (2017) outlined that unfinished goals would be highly accessible in memory and interfere with task performance (Masicampo and Baumeister, 2011a,b). Weigelt et al. (2018) expanded this assumption by building on the perspective of stress-as-offense-to-self (cf. Semmer et al., 2019 for recent description). They found that unfinished tasks were linked to a lowered self-evaluation of feeling competent, indicating a threat to one’s self-image. This was associated with higher affective rumination. In summary, the higher the number of unfinished tasks, the higher the discrepancy between the desired and current states. The higher the discrepancy, the lower the possibility of overcoming it (especially in the case of negative self-evaluation, see Watkins, 2008; Weigelt et al., 2018), with a higher probability of negative connoted rumination. Furthermore, the higher the discrepancy, the harder it is to abandon and disengage from the goal at hand or to switch to more helpful and concrete thought processes (cf. Watkins, 2008). Studies evaluating weekends confirmed the association between unfinished tasks and affective rumination (Syrek and Antoni, 2014; Syrek et al., 2017; Weigelt et al., 2018). Therefore, it can be concluded that unfinished tasks would be associated with a higher level of affective rumination on a daily level. Furthermore, higher average levels of unfinished tasks would also be associated with higher average levels of affective rumination.

*Hypothesis 1: More unfinished tasks in the afternoon will be associated with higher affective rumination in the evening at (a) the within- and (b) the between-person levels.*

Moreover, fatigue should be related to higher levels of affective rumination. Recent empirical findings regarding reciprocal relationships between low psychological detachment and its expected outcomes demonstrate that higher strain levels may influence ruminative thoughts in the evening (Jimenez et al., 2022; Wendsche et al., 2021). Fatigue is a state common to human life and is often characterized by low mood, unfocused mental states, or unpleasant bodily states (Hockey, 2013). It is often linked to mostly uncomfortable effects and undesirable deactivation (Yik et al., 2011; Bennett et al., 2018). In this study, fatigue was understood as a state of subjectively feeling tired and exhausted (Hockey, 2013). Therefore, it constitutes the late stage of a process in which sustained effort develops into an aversive state (Hockey, 2013). It is assumed that fatigue varies within and between persons (cf. Sonnentag et al., 2014; Bennett et al., 2018).

Following the recovery paradox (Sonnentag, 2018), higher levels of fatigue should make affective rumination more likely for two reasons. First, Sonnentag (2018) argued from an energy depletion perspective and stated that feeling exhausted and fatigued would lead to difficulties in controlling emotional reactions and thought processes about work. Therefore, feeling fatigued and exhausted should be associated with higher affective rumination as a means of depleted energy resources. Second, fatigue is characterized as an unpleasant and negative state (Yik et al., 2011; Hockey, 2013). Sonnentag (2018) argued on the basis of the mood-congruency hypothesis (Bower, 1981; Judge and Ilies, 2004) and suggested that negative affective states after work would increase the accessibility of negative cognitions about work.

The notion of fatigue, interpreted as depletion of energy, has recently been challenged from a control theory or self-regulation perspective (Hockey, 2013; Inzlicht et al., 2014, 2021). From this perspective, fatigue is considered as an emotion that has adaptive functions to prevent one from being fixated on current activities and to shift attention toward activities with higher utility (Inzlicht et al., 2014). Fatigue supposedly functions “to alert the organism to both the costs of persisting with effortful, unrewarding activities and the benefits of engaging with more rewarding ones, and thus maintain effective motivational equilibrium” (Hockey, 2013, p. 104). This is more likely when negatively attributed work stressors are high, and effort is needed to maintain attention on work goals (Hockey, 2013). Fatigue might increase the salience of the “want-to” goal of leisure time, but also of the costs of the unrewarding activity (“have-to” goal, cf. Inzlicht et al., 2014), which is associated with fatigue in the afternoon (e.g., a stressful day). Work is generally presented daily. As the fatiguing activity and costs that accompany it will be present the next day (or after the weekend), negatively connoted work-related thoughts would be present when feeling highly fatigued. A concrete context and real-life work example can be considered in the educational sector. Specifically, one could imagine a tired and fatigued teacher thinking about a student, who constantly behaves disruptively in class and makes work for this teacher more stressful and less satisfying. Feeling exhausted might result in the teacher mentally connecting work with fatigue (cf. Meurs and Perrewé, 2011) and contemplate the high costs of work and the inability to experience work as being pleasant and fulfilling the next day (leisure, “want-to”; e.g., Inzlicht et al., 2014). Thus, the discrepancy between the “want-to” goal (experiencing work satisfaction, pleasure due to leisure time) and their actual state is high, while the control to overcome the discrepancy is lowered, which makes unfavorable ruminative thoughts more probable. The occurrence of affective rumination might further be fostered by mood-congruent memory processes, as proposed by Sonnentag (2018; see also Watkins, 2008). Therefore, higher fatigue should be related to higher affective rumination. This is consistent with the recovery paradox; that is, it is more difficult to recover when it is most needed (Sonnentag, 2018; Wendsche et al., 2021). Therefore, lower psychological detachment and higher affective rumination should not only lead to fatigue and exhaustion, as indicated by meta-analyses (e.g., Steed et al., 2021), but should also be predicted by fatigue and exhaustion, which is described as a reversed effect. Empirical evidence from cross-lagged studies focusing on between-person level effects supports that higher exhaustion predicts lower levels of psychological detachment (Sonnentag et al., 2014; Schulz et al., 2020). Kinnunen et al. (2019) showed that lower vigor, as one facet of work engagement, predicted higher affective rumination in a cross-lagged longitudinal study. They found no support for a significantly better fit for the reversed model when studying the effect of emotional exhaustion on affective rumination when compared to the model without reversed effects. However, they still concluded that “reversed effects gained most support” (Kinnunen et al., 2019, p 569).

Based on the above argument and the encouraging, but unclear findings, we argue that the more a person experiences fatigue, the more affective rumination will be present. Furthermore, it is hypothesized (cf. Meurs and Perrewé, 2011; Wendsche et al., 2021) that this will be present on the daily (within-person) and average (between-person) levels.

*Hypothesis 2: Higher fatigue levels in the afternoon will be associated with higher affective rumination in the evening at (a) the within- and (b) the between-person levels.*

As indicated, both lines of research concerning unfinished tasks as well as fatigue as antecedents of affective rumination can be explained and argued from a control theory or self-regulation perspective (Watkins, 2008; Carver and Scheier, 2016; Inzlicht et al., 2021). We propose that both fatigue and unfinished tasks will explain different parts of variance in affective rumination for the following reasons: unfinished tasks should act as an external stimulus for goal discrepancies between one’s goal to finish work and the actual state of having unfinished tasks. It might further function as an internal stimulus for experiencing a threat to one’s self (Weigelt et al., 2018). Fatigue may also act as an internal stimulus of poor goal progress and high costs of activities. This can be exemplified as, a day of high workload, which is present the next day, with little intrinsic value, while highlighting other goals of higher utility (Hockey, 2013; Inzlicht et al., 2014). Thus, the discrepancy would be between the actual state of high costs of work and pleasurable affect due to intrinsic valued goals, such as experiencing leisure time and control over one’s activities. Considering various stimuli as reference values for different goals and motives (cf. Watkins, 2008), we argue that unfinished tasks and fatigue might share variance but should explain unique shares of variance in affective rumination.

*Hypothesis 3: Higher fatigue levels and more unfinished tasks in the afternoon will explain variance of higher affective rumination in the evening at (a) the within- and (b) the between-person levels.*

### Prediction of Vitality: Cyclical Processes

Vitality is described as feeling alive and energetic and is conceptualized as being influenced by one’s fatigue level (Ryan and Frederick, 1997; Fritz et al., 2011). Ryan and Frederick (1997) noted that “to the degree that one is free of conflicts, unburdened by external controls, and feeling capable of effecting action, then one should report higher vitality” (Ryan and Frederick, 1997, p. 530). In contrast, “conflicts and demands on the self that threaten self-regulation and actualization, particularly those associated with feeling a lack of effectance, autonomy, or relatedness, are expected to diminish vitality” (Ryan and Frederick, 1997, p. 531; see e.g., Kleine et al., 2019). This definition is similar to that described above regarding fatigue (Hockey, 2013).

Watkins (2008) suggested that negatively valenced thought processes are associated with unfavorable outcomes. Furthermore, following Brosschot et al. (2005) and Meurs and Perrewé (2011), perseverative cognitions (e.g., rumination) might best explain the duration of stressful reactions after experiencing work stressors, which were coupled with negative expectations. For instance, in our example, the disrupting student will be present at school the next day. Moreover, mental representations of stressful experiences (e.g., thoughts about the student) would prolong stressful experiences. Similarly, Sonnentag and Fritz (2015) argued that negative thoughts during non-work time would keep work present in one’s mind and interfere with a healthy recovery experience. Therefore, affective rumination should interfere with recovery, restrict overcoming goal discrepancies, and increase fatigue levels. Thus, it is likely that the higher the level of affective rumination, the lower the probability of feeling vital the next day (cf. also Hockey, 2013). As lower vitality should lead to increased efforts to protect goal attainment at work and to further fatigue in the afternoon (cf. Hockey, 2013), the effect of affective rumination should be present at the within- and between-person levels.

Minnen et al. (2021), for example, showed that affective rumination was related to higher fatigue and lower vigor, which was defined as higher levels of emotional, cognitive, and physical energy (all measured simultaneously). Following this and the above argument, we proposed that affective rumination in the evening would be related to lower levels of vitality the next morning (cf. Firoozabadi et al., 2018b) at the between- and within-person levels.

*Hypothesis 4: Higher affective rumination in the evening is related to lower vitality the next morning at (a) within-person and (b) between-person levels.*

**FIGURE 1:**
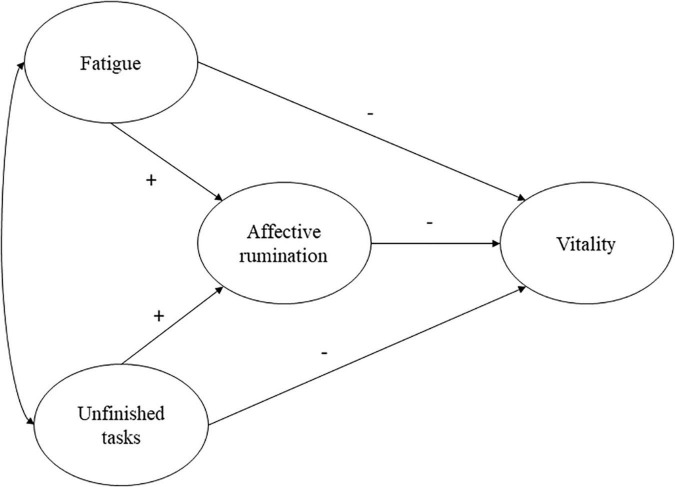
Hypothesized model at the between- and within-person levels.

Moreover, unfinished tasks in the afternoon have been related to higher affective rumination. As argued above, this should lead to lower vitality the next morning. Following Brosschot et al. (2005); Meurs and Perrewé (2011), and Hockey (2013), work-related rumination in the form of affective rumination should prolong one’s stress experience as well as fatigue level. Therefore, a cyclical process is tested, in which fatigue predicts vitality the next morning via affective rumination. The proposed model is illustrated in Figure 1.

*Hypothesis 5: More unfinished tasks in the afternoon are related to lower vitality the next morning at (a) within-person and (b) between-person levels via affective rumination.*

*Hypothesis 6: A higher fatigue level in the afternoon is related to lower vitality the next morning on (a) within-person and (b) between-person levels via affective rumination.*

## Materials and Methods

We selected beginning teachers in Germany as participants, whom, after graduating from university, have to finish an induction phase before being qualified as a teacher (cf. Voss and Kunter, 2020). During the induction phase, beginning teachers are placed at schools and teach in class. They are supervised by experienced teachers and attend seminars on general teaching methods (cf. Voss and Kunter, 2020). While this system might differ to educational systems in other countries or other occupations, the first years of teaching are considered especially difficult and proposedly include a “reality shock,” which is characterized by feelings of high strain (cf. Dicke et al., 2015, 2018). Furthermore, teachers are especially likely to experience work-related thoughts during non-work times, and a recent systematic review advocated for further investigating this sample (Türktorun et al., 2020). Therefore, this sample is especially suitable for studying work-related thoughts during non-work time.

### Sample and Procedure

The beginning teachers were recruited from different schools in Hesse, Germany, between October 2019 and March 2020. First, an official invitation from the Hessian Teacher Academy was sent to the beginning teachers in Hesse. Second, beginning teachers were invited using a snowballing technique. The study adhered to ethical standards as the beginning teachers were informed about the procedure, data privacy policies, and that the data would only be used for research purposes. Furthermore, participation was voluntary after written consent and canceling participation was possible at any time without detriments. As an incentive, the participants received vouchers for an online retailer and feedback on the study results. In total, 80 beginning teachers agreed to participate.

We used an online survey platform to allow participation to utilize digital devices. The participants were asked to complete a mandatory online general questionnaire, which included a survey of demographic and work-related variables. One week later, the participants received an invitation to the daily questionnaires conducted from Wednesday to Monday morning (including 3 weekdays and the weekend), three times a day. In the late afternoon after work (03:00–06:15 p.m.), they answered questions about their fatigue level and unfinished tasks. In the late evening (08:30–11:30 p.m.), they indicated their level of affective rumination. The participants were asked to indicate their level of vitality in the morning before work (05:00–08:30 a.m.). As unfinished tasks were a main focus of the present study, only days on which participants worked were included in the data analyses. This was indicated via an affirmation in the afternoon or (when participants missed the afternoon survey) in the evening that the participant had worked. These days could be characterized by working time at school, in a seminar or at home. Furthermore, only those participants who worked at least twice during the study period were included, as within-person variance was a central part of the study. Following this premise, five beginning teachers were excluded from the study. One beginning teacher participated during the lockdown period in Germany (starting from the 16th of March) and was excluded. The final sample consisted of 74 beginning teachers (female = 78.4%; male = 21.6%, *M* = 28.47 years old, *SD* = 4.69). The overrepresentation of female participants is consistent with statistics in Hesse, Germany, where for example over 71% of primary and secondary school teachers are female (cf. Hessisches Statistisches Landesamt, 2020). The beginning teachers worked in different school types. A total of 274 daily measures in the afternoon (missing data: 2.49%), 252 in the evening (missing data: 10.32%), and 269 in the morning (missing data: 4.27%) were collected. All working days (*n* = 281) comprised of afternoon, evening, and next morning measures were included, even those with missing data (Grund et al., 2019; cf. Hox, 2010). The average cluster size, which refers to the average amount of working days clustered within the beginning teachers, was 3.8 days.

### Measures

Table 1 shows the descriptive statistics and correlations of the scales used. IBM SPSS Statistics (Version 27) and MPlus6.1 (Muthén and Muthén, 1998-2010) were used to analyze descriptive statistics.

**TABLE 1:** Descriptive statistics and correlations of level 1 variables.

Variables	*M*_*b*_	*SD*_*b*_	ICC	1	2	3	4
1 Affective rumination	2.41	0.73	0.597		0.202**	0.199**	–0.047
2 Unfinished tasks	2.65	0.70	0.415	0.555**		0.140^†^	–0.096^†^
3 Fatigue	2.48	0.52	0.277	0.568**	0.462**		–0.04
4 Vitality	3.13	0.51	0.336	–0.640**	–0.389**	–0.576**	

*For the correlations, standardized coefficients are presented. The coefficients are based on random intercept models. Within-person level correlations are shown above the diagonal. Between-person level correlations are shown below the diagonal. n_level2_ = 73–74. n_level1_ = 252–281. M_b_/SD_b_ = estimated between-person level mean and standard deviation. ICC, Intraclass Correlation. Unfinished tasks and fatigue were measured in the afternoon (03:00–06:15 p.m.). Affective rumination was measured in the late evening (08:30–11:30 p.m.). Vitality was measured in the morning before work (05:00–08:30 a.m.).*

^†^p < 0.10, one-tailed. **p < 0.01, one-tailed.

#### Affective Rumination

The German version (cf. Hamesch et al., 2014) of the Work-Related Rumination Questionnaire (Cropley et al., 2012) was used to assess affective rumination with five items on a five-point Likert scale ranging from 1 (*strongly disagree*) to 5 (*strongly agree*). The items were slightly adapted to the daily context so that the items started with “This evening…I became tense when I thought about work-related issues during my free time.” Cronbach’s alpha for the 5 days ranged from 0.822 to 0.898.

#### Fatigue

In the afternoon, fatigue was assessed using four items of the German version of the Profile of Mood States (Albani et al., 2005). The beginning teachers indicated their fatigue level using rating adjectives (e.g., “exhausted”) on a five-point Likert scale ranging from 1 (*not at all*) to 5 (*extremely*). Cronbach’s alpha for the 5 days ranged from 0.875 to 0.921.

#### Unfinished Tasks

Unfinished tasks were assessed using a six-item scale developed by Syrek et al. (2017) that was slightly adapted to the daily context. The items were assessed after school and answered on a five-point Likert scale from 1 (*completely inapplicable*) to 5 (*completely applicable*). A sample item was “Today I have not finished important tasks that I had planned to do” (see also Syrek and Antoni, 2014; Weigelt et al., 2018). Cronbach’s alpha for the 5 days ranged from 0.873 to 0.928.

#### Vitality

Three items of the German version (Fritz et al., 2011) of the Subjective Vitality Scale developed by Ryan and Frederick (1997) were used to assess vitality in the morning. The participants were asked to answer the items according to their present state on a Likert scale ranging from 1 (*strongly disagree*) to 5 (*strongly agree*). A sample item was “I feel alive and vital.” Cronbach’s alpha for the 5 days ranged from 0.903 to 0.944.

### Data Structure and Analysis

The final sample included 74 participants, with 281 working days. The sample size was well above 40, and convergence problems were not expected (cf. Li and Beretvas, 2013). The days included fatigue and unfinished tasks assessed in the afternoon after work, affective rumination assessed in the evening, and vitality assessed the next morning. To use all available data, all working days with at least one assessment were included. Full information maximum likelihood estimation was used to manage the missing data (cf. Grund et al., 2019). The intraclass correlation, which provides information on the variance components of the study variables, is shown in Table 1. All predictor variables were grand mean centered. To check whether they were separate constructs (see Hypothesis 3), a multilevel confirmatory factor analysis (MCFA) with the MLF estimator was conducted using Mplus6.1 (Muthén and Muthén, 1998-2010). For the unfinished tasks scale, it was necessary to constrain one residual variance of one item to zero (Muthén, 2005, 20 January) on the between-person level, to deal with two items that had a negative residual variances in two separate MCFAs. All analyses were conducted with a four-item unfinished task scale (excluding both items), which did not lead to decisively different results. A six-item scale was used to maintain comparability between studies (e.g., Syrek et al., 2017). The MCFA with a one-factor solution led to a poor fit to the data, χ^2^(71) = 778.01, *p* < 0.001, comparative fit index (CFI) = 0.592, Tucker-Lewis index (TLI) = 0.482, root mean square error of approximation (RMSEA) = 0.191, standardized root mean square residual within (SRMR_W_) = 0.239, standardized root mean square residual between (SRMR_B_) = 0.309. A two-factor solution with correlated factors for fatigue and unfinished tasks had an acceptable fit (Schermelleh-Engel et al., 2003) to the data: χ^2^(69) = 136.23, *p* < 0.001, CFI = 0.961, TLI = 0.949, RMSEA = 0.06, SRMR_W_ = 0.043, SRMR_B_ = 0.094. The model had a significantly better fit to the data than the one-factor solution χ^2^_Δ_ (2) = 641,78, *p* < 0.001. Therefore, we accepted the two-factor solution, and concluded that both constructs were distinct.

The hypotheses were tested using an overall multilevel path model using Mplus6.1 with the MLR estimator (Muthén and Muthén, 1998-2010) in which between-person and within-person level associations were estimated simultaneously. The within-person level corresponds to implicitly group-mean-centered variables (Preacher et al., 2010). The model corresponded to a random-intercept fixed-slope model to reduce complexity (cf. Preacher et al., 2010). Unfinished tasks and fatigue were allowed to correlate. Indirect effects were tested by estimating 90% confidence intervals (90% CI) with Monte Carlo simulations (Preacher et al., 2010) using the web-based interactive tool by Selig and Preacher (2008). Monte Carlo simulations with 20,000 repetitions were conducted (also called Monte Carlo method or parametric bootstrap, cf. Preacher et al., 2010). A 90% CI is suitable in the mediation context and in the case of one-tailed hypothesis testing (Preacher et al., 2010). The 90% CIs are given for unstandardized point estimations. However, standardized values are presented for all other estimations. Recently, controlling for (cyclical) trends of variables within diary studies has been stressed upon (Gabriel et al., 2019; Sonnentag et al., 2021). This is because trends might exist, which capture changes in the variables due to certain factors such as a particular work context or cultural factors (Gabriel et al., 2019). There is evidence, for example, that the levels of fatigue (and therefore vitality) are related to the weekday (Zijlstra and Rook, 2008). To control for fluctuations of vitality and affective rumination in dependence of the weekday, hypothetical linear, sine, and cosine functions for the corresponding 5 days were tested as predictors and control variables at the within-person level (see Gabriel et al., 2019; Sonnentag et al., 2021). A sine wave “captures growth that increases from a baseline to a peak, followed by decreasing growth to a trough and then returns to the baseline” (Gabriel et al., 2019, p. 984). A cosine wave “follows the same pattern but begins with decreasing growth” (Gabriel et al., 2019, p. 984). The mentioned sine and a cosine function variables were created based on Gabriel et al. (2019) suggested formula, which captures a sine and cosine wave over the week (Gabriel et al., 2019, p. 984). As the study started on a Wednesday the values of the sine and cosine wave for the third to seventh day was calculated based on the formula by Gabriel et al. (2019). In detail, a significant positive association with a linear trend would indicate a linear growth of vitality or affective rumination within the beginning teachers over the days of the study. A significant positive association with the sine wave would indicate a change in vitality or affective rumination, which decreases the first 3 days and increases the next 2 days. A significant positive association with a cosine function would indicate a change following a cosine wave, which means an increase in the variables over the days of the study.

## Results

### Results of the Linear and Cyclical Trends

In the first step, the linear and sine functions were simultaneously included as predictors of affective rumination and vitality at the within-person level. Both models are listed in Table 2. The linear and cosine function did not predict vitality or affective rumination. Only the sine function predicted affective rumination and in tendency, vitality (*p* = 0.107) and it was included as a control variable in the model below. As the association with affective rumination was positive, this indicates that affective rumination might slightly follow a sine wave over the course of the study as described above. A negative association with vitality would indicate that vitality might slightly follow the opposite direction of the sine wave as described (an increase over the first days, followed by a decrease).

**TABLE 2:** Results of the linear and cyclical trends.

	Vitality	Affective rumination
	
Parameter	Estimate (SE)	Estimate (SE)
Linear	0.013 (0.196)	–0.057 (0.181)
Sine	–0.273 (0.169)	0.341 (0.155)*
Cosine	0.169 (0.330)	0.036 (0.325)

*Standardized coefficients are presented. The two models show within-person level results as the parameters were defined on the within-person level. Model with fatigue as dependent variable: n_level2_ = 74. n_level1_ = 269. Model with affective rumination as dependent variable: n_level2_ = 73. n_level1_ = 252.*

*p < 0.05, two-tailed.

### Test of Hypothesis

Table 3 lists the standardized coefficients of the multilevel path model. In line with Hypotheses 1a, 1b, 2a, 2b, and 3, unfinished tasks and fatigue in the afternoon both predicted affective rumination in the evening. This was true for the between-person and within-person levels. Higher average levels of unfinished tasks and higher levels of fatigue were related to higher average levels of affective rumination (between-person level). Furthermore, higher daily unfinished tasks and fatigue were related to higher daily affective rumination (within-person level).

**TABLE 3:** Correlations and estimates of predictors in the multilevel path model.

	Vitality	Affective rumination
	
	Estimate (SE)	Estimate (SE)
**Between-person level**		
Fatigue	–0.362 (0.221)^†^	0.384 (0.191)*
Unfinished tasks	0.017 (0.189)	0.375 (0.16)**
Affective rumination	–0.444 (0.184)**	–
Residual variance	0.505 (0.163)**	0.575 (0.140)**
R-square (between)	0.495 (0.163)**	0.425 (0.14)**
**Within-person level**		
Fatigue	0.007 (0.07)	0.149 (0.061)**
Unfinished tasks	–0.073 (0.07)	0.163 (0.073)*
Affective rumination	0.001 (0.076)	–
Sine	–0.183 (0.078)*	0.175 (0.069)**
Residual variance	0.959 (0.03)**	0.901 (0.043)**
R-square (within)	0.041 (0.03)^†^	0.099 (0.043)*

*Standardized predictor coefficients are presented. n_level2_ = 74. n_level1_ = 281. Unfinished tasks and fatigue were not significantly related on the within-person level, r = 0.135, SE = 0.091, p = 0.067, but significantly related on the between-person level, r = 0.472, SE = 0.168, p = 0.003. R-Square estimations were calculated in Mplus6.1 (Muthén and Muthén, 1998-2010; Muthén, 2015, 14 October). These estimations are based on Snijder and Bosker (1999) formula regarding R-Square for explained variance on level 1 (within) and level 2 (between). Fatigue and unfinished tasks were assessed in the afternoon (03:00–06:15 p.m.). Affective rumination was assessed in the late evening (08:30–11:30 p.m.). Vitality was assessed in the morning before work (05:00–08:30 a.m.).*

^†^p < 0.10, one-tailed. *p < 0.05, one-tailed. **p < 0.01, one-tailed.

Contrary to Hypotheses 4a, 5a, and 6a, neither affective rumination and fatigue nor unfinished tasks predicted vitality the next morning at the within-person level. Only the sine function was a significant negative predictor, indicating that vitality might change as a function of the days of the study following the opposite of a sine wave within the beginning teachers. Following these results, there was no indirect effect of fatigue, *b* < 0.001, 90% CI [–0.02, 0.02] on vitality, and no indirect effect of unfinished tasks, *b* < 0.001, 90% CI [–0.02, 0.02] on vitality via affective rumination. Hypotheses 5a and 6a were not supported.

At the between-person level, higher affective rumination was related to lower vitality the next morning. Thus, Hypothesis 4b was supported. There was also a tendency for higher fatigue in the afternoon to be related to lower vitality the next morning. However, this effect failed to reach significance (*p* = 0.051). Furthermore, indirect effects were tested following Hypotheses 5b and 6b. The indirect effect of fatigue in the afternoon on vitality the next morning via affective rumination was significant, b = –0.17, 90% CI [–0.36, –0.01]. The indirect effect of unfinished tasks in the afternoon on vitality the next morning via affective rumination was significant, b = –0.12, 90% CI [–0.27, –0.01]. Affective rumination fully mediated the relationship between unfinished tasks and vitality, as the direct path of unfinished tasks on vitality (cf. Table 3) was not significant in the model.

## Discussion

The aim of the present study was to bridge and integrate current theories on what causes affective rumination during non-work time (cf. Wendsche et al., 2021). Specifically, unfinished tasks and fatigue were considered as predictors of affective rumination, and it was deduced that the recovery paradox phenomenon (Sonnentag, 2018) and self-regulation model of ruminative thoughts (Martin and Tesser, 1996) are integrable. Furthermore, cyclical or reciprocal processes for fatigue and vitality with affective rumination as mediators were tested.

### Theoretical Contributions

The present study emphasizes that when not being able to finish their tasks and experiencing fatigue at the end of the workday, beginning teachers tend to experience affective rumination. The findings are significant as the associations were found over the course of 5 days (between-person level) as well as when participants experienced more unfinished tasks and fatigue than usual (within-person level). Thus, the results indicate that daily changes in unfinished tasks and fatigue were associated with daily changes in affective rumination. Furthermore, there were differences between persons experiencing fatigue and unfinished tasks and accompanying affective rumination. More crucially, the findings show that different antecedents may be equally important, and different processes might explain why affective rumination occurs. The abovementioned results are consistent with the recovery paradox; namely, that it is most difficult to recover when it is most needed (Sonnentag, 2018; Wendsche et al., 2021). Furthermore, we argued that the results can be explained from a control theory perspective. Unfinished tasks might refer to unfinished goals referring to work (external) and might act as a threat to one’s competence need satisfaction (internal), as suggested by Weigelt et al. (2018). Conversely, fatigue might refer to the need to focus on more intrinsic valued goals (internal) and high costs of work (external). As fatigue is considered an unpleasant affect, mood-congruent memory processes may strengthen the possibility of negatively valenced thoughts (cf. Sonnentag, 2018). Therefore, affective rumination may have different antecedents according to the discrepancies in different goals. This is consistent with the notion of Martin and Tesser (1996), who suggested that similar behavior might be observable due to different underlying goals (or goal discrepancies) and internal or external triggers (equifinality). Therefore, the results contradict the assumption that there is one central stimulus for the occurrence of affective rumination, such as work stressors, or internal stimuli, such as fatigue or threats to one’s self-esteem. This is because they might be equally associated with affective rumination in different situations. The results suggest the need to further differentiate the predictors and antecedents of affective rumination.

Moreover, the present study highlights that having many unfinished tasks and feeling fatigued and tired in the afternoon are related (see Table 1) but are not necessarily interlinked. This can be regarded from a control theory perspective (Martin and Tesser, 1996; Watkins, 2008). On the one hand, a higher discrepancy between one’s goal of task completion and the present state of having unfinished tasks may be associated with higher fatigue as an indicator of the costs of reducing goal discrepancies. This might be strengthened when there is a low possibility of reducing the discrepancy observed in that evening (cf. Meurs and Perrewé, 2011; Hockey, 2013). Furthermore, the relationship at the between-person level is consistent with Hockey’s (2013) findings. He suggested that fatigue would lead to increased effort to focus on and finish important unattained work goals, which would subsequently lead to increased fatigue. Therefore, over the course of 5 days, more unfinished tasks are likely to be related to fatigue levels. Sonnentag et al. (2014) further suggested that feeling more fatigued would decrease the ability to complete tasks at work. In contrast, having many unfinished tasks might motivate employees to work in the evening to overcome the discrepancy and therefore experience a lower level of fatigue (cf. Weigelt and Syrek, 2017).

We hypothesized that lower levels of affective rumination are related to feeling alive and vital. While this held true over the course of 5 days, it was not observed at the within-person level. Therefore, reciprocal processes may be present at the between-person level, and not at the within-person level. A higher average level of fatigue is related to affective rumination, which is associated with vitality at the between-person level. These results are consistent with the findings which show that exhaustion can be a predictor of low psychological detachment (e.g., Sonnentag et al., 2014). However, they contradict Sonnentag et al. (2021), who found cyclical processes for positive work reflection at the within-person level but not at the between-person level. The results are unexpected, as within-person level analyses of affective rumination had formally demonstrated relationships with outcomes such as sleep, wellbeing, or vigor (Syrek et al., 2017; Firoozabadi et al., 2018b; Wach et al., 2020; Minnen et al., 2021). Notably, the results cannot be explained by low levels of within-person variance in the constructs, as all constructs had within- and between-person variance (see Table 1). However, daily level studies in which the outcome is measured on the next day are still scarce (Firoozabadi et al., 2018b; Wach et al., 2020). Moreover, differences between within-person and between-person level relationships were also found for other wellbeing indicators (McCormick et al., 2020). For example, McCormick et al. (2020) showed that relationships pertaining to happiness differed between both levels of analyses. They implied “that the causes and consequences of an employee being happy at a given time are different from the causes and consequences of some employees being generally happier than others” (McCormick et al., 2020, p. 339). One explanation might be deduced from the research on depressive rumination. Watkins (2008) pointed out that depressive rumination only negatively impacted individuals with an elevated level of negative life events and more depressed mood as well as more negative self-beliefs and more pessimistic attributions. As experiencing higher levels of affective rumination appeared in the context of feeling more fatigued in general and having more unfinished tasks, this could be the context in which affective rumination negatively influences vitality. Higher affective rumination than usual (within-person level) might be less detrimental for feeling vital the next morning.

An interesting finding, which was not the focus of the study, was that vitality and affective rumination might fluctuate within a person over the days of the study. This was shown in the prediction of affective rumination and vitality by the sine function, which was significant for affective rumination and implicated in tendency for vitality (see first model, *p* = 0.107, vs. second model). This is consistent with the growing research on weekday effects on fatigue, which focused on cycles of stress experience, mood, and fatigue depending on the weekday (e.g., Rook and Zijlstra, 2006; Nicholson and Griffin, 2017; Pindek et al., 2020). The present results contribute to the research, as it shows that the sine function of the weekday is related to vitality. It extends the research by adding a relationship of the weekday to further internal thoughts and feelings, such as affective rumination. As Sonnentag et al. (2021) did not find such processes for positive work reflection, this might only hold true for negative connoted forms of work-related feelings and thoughts. However, we only considered days in which the participants worked, and it is unclear whether this relationship will be observed on weekends or on weekdays when participants do not work.

### Study Limitations and Future Research Implications

The present study has limitations; however, it also has implications for future research. First, affective rumination was considered as the operationalization of work-related rumination and negative connoted work-related thoughts as well as feelings. Rumination contains not only thought processes, but also other mental representations, such as images, and is accompanied by emotions (cf. Martin and Tesser, 1996); thus, considering affective rumination is justifiable. The differentiation between the abstractness or concreteness of the content of thought processes was not considered. Watkins (2008) showed that negative valenced thoughts have more maladaptive outcomes when being abstract (focusing on the “why”) compared to being more concrete. In contrast, positive thoughts are more helpful when abstract, rather than concrete. The difference in the abstractness of positive thoughts and its effect is apparent in Sonnentag et al. (2021) compared to Flaxman et al. (2017). However, further research is required regarding the negative connoted forms of work-related thought processes. Second, the study design did not allow for conclusions to be drawn on causality in the relationships tested. Experimental designs are necessary to obtain causal conclusions (cf. Sonnentag et al., 2021). For example, Huffziger et al. (2012) introduced young adults to the utilization of palmtop computers to ruminate and contemplate their current feelings and consequences. They found a short-term increase in ruminative self-focus and a decrease in calmness. Transferred to work context, one could consider an experiment in which participants are asked to think about their work, or even positive vs. negative situations at work, to induce work-related thought processes. Furthermore, inducing fatigue or manipulating the level of unfinished tasks can also (Zeigarnik, 1938; Hockey, 2013) increase causality in the aforementioned relationship. These studies would increase the possibility of causal inferences and would be a valuable addition to current diary research on work-related rumination. Third, the study focused on beginning teachers and, accordingly, observed a group of employees with less work experience. While teachers form an important sample for work-related rumination studies (Türktorun et al., 2020; Varol et al., 2021), this might reduce the generalizability of the results. One might consider unfinished tasks as an especially important predictor in inexperienced employees and novice teachers who lack the resources to quickly overcome discrepancies caused by unfinished tasks, and more experienced employees might not ruminate about the unfinished tasks at hand. The effects of unfinished tasks have been demonstrated in numerous studies (Syrek et al., 2017; Weigelt et al., 2018). However, it remains important to test work experience as a possible moderator in future research. Fourth, one important aspect is that fatigue might influence affective rumination, especially under work conditions. We did not test whether the relationship between fatigue and affective rumination was still present on non-workdays or on vacations. This should be especially interesting in teachers who have longer vacation times in Germany and endeavor to complete a significant amount of work before vacations. Therefore, future research might examine the differences in this relationship during vacations.

Furthermore, there was a tendency for affective rumination to not fully mediate the relationship between fatigue in the afternoon and vitality in the morning at the between-person level (*p* = 0.051). There might be further mediating factors, which could explain why fatigue in the afternoon is related to vitality at the between-person level. For example, fatigue might lead to difficulties in physical recovery experiences (Sonnentag, 2018), which might reduce the overall feeling of vitality and aliveness.

Additionally, the moderating factors between work-related thoughts and outcomes were not considered. Self-control and self-regulation have mostly been investigated (Firoozabadi et al., 2018b; Junker et al., 2020), which makes sense since affective rumination is associated with lowered executive functioning (Cropley et al., 2016; Cropley and Collis, 2020). Factors that moderate the relationship between fatigue or unfinished tasks and affective rumination remain unaddressed in recent research as well as the present study. For example, Watkins and Nolen-Hoeksema (2014) hypothesized that depressive rumination would be a habit of reacting to negative affect. Therefore, trait rumination might moderate the relationship between fatigue and affective rumination. Furthermore, the centrality of work to one’s identity (cf. Kossek et al., 2012) may be an important factor. As unfinished tasks might constitute a threat to one’s self-esteem, which might foster affective rumination (Weigelt et al., 2018), this should be prevalent among individuals with higher centrality of work to one’s identity (cf. Martin and Tesser, 1996).

### Practical Implications

The present study adds to previous research that shows the negative impact of affective rumination on wellbeing indicators (cf. Jimenez et al., 2022). It further adds to extant research by demonstrating that participants, who had a higher overall level in ruminating, showed harmful associations. Therefore, those particular employees might benefit from interventions to reduce their affective rumination (cf. Karabinski et al., 2021). Some interventions in occupational health psychology focus on reducing rumination or increasing distancing oneself from work in the evening (see also McCarrick et al., 2021). Karabinski et al. (2021) showed in their meta-analysis that interventions focusing on boundary management, emotion regulation, and sleep improvement were effective in increasing detachment from work (with moderate effect sizes). Additionally, interventions with training in mindfulness, problem-focused coping, or engagement in active recovery activities were effective (Karabinski et al., 2021). However, interventions on work (e.g., reducing job demands) were less likely to be studied, suggesting that more research is needed for effective interventions at the occupational level.

We conducted the study with beginning teachers. Supervisors, who accompany beginning teachers, and seminar lecturers, should focus on integrating content concerning how to structure work in the early stages of their practical career. Furthermore, as fatigue level is an important antecedent of affective rumination, beginning teachers should be strongly encouraged to invest in recovery experiences (cf. Karabinski et al., 2021) instead of continuously and constantly increasing effort with potentially harmful long-term effects (cf. Hockey, 2013).

Finally, the study shows that various internal and external stimuli are important, which might explain the occurrence of affective rumination. Martin and Tesser (1996) contended that it is important to attain or abandon a goal to reduce ruminative thoughts. Following this notion, to reduce affective rumination, one must be aware of the current antecedent of affective rumination and its related needs (higher hierarchy goal). For example, affective rumination due to unfinished tasks might be managed by becoming aware of one’s resources (Weigelt et al., 2018), such as social support from colleagues or supervisors, or in planning the task execution (Smit, 2016; Smit and Barber, 2016). However, affective rumination due to fatigue might be prevented by using active recovery or boundary management strategies while focusing on an intrinsic valued goal (Hockey, 2013; Inzlicht et al., 2014; Karabinski et al., 2021).

## Conclusion

The present study focused on fatigue and unfinished tasks as antecedents of affective rumination and the association between affective rumination in the evening and vitality the next day among beginning teachers. Findings revealed that both unfinished tasks and fatigue were predictors of affective rumination in the evening. Hence, different antecedents are important. Furthermore, affective rumination is an important factor in recovery research, as experiencing higher affective rumination on average is related to lower average levels of vitality. However, this might only be true at the between-person level, as days with higher affective rumination is not necessarily associated with lower vitality within persons. Therefore, this study adds to the research emphasizing the importance of affective rumination while encouraging further study and interpretation of the antecedents of affective rumination from a control theory or self-regulation theory perspective.

## Data Availability Statement

The datasets presented in this article are not readily available because the participants were guaranteed that the data would not be passed on to third parties. Requests to access the datasets should be directed to GW.

## Ethics Statement

Ethical review and approval was not required for the study on human participants in accordance with the local legislation and institutional requirements. The participants provided their written informed consent to participate in this study.

## Author Contributions

GW and YV designed the study and responsible for data collection and data management. HH supervised the design process. GW had the main responsibility for writing this manuscript and conducting statistical analysis while YV and HH helped reviewing and editing the manuscript. All authors contributed to the article and approved the submitted version.

## Conflict of Interest

The authors declare that the research was conducted in the absence of any commercial or financial relationships that could be construed as a potential conflict of interest.

## Publisher’s Note

All claims expressed in this article are solely those of the authors and do not necessarily represent those of their affiliated organizations, or those of the publisher, the editors and the reviewers. Any product that may be evaluated in this article, or claim that may be made by its manufacturer, is not guaranteed or endorsed by the publisher.
